# Balancing Water Uptake and Loss through the Coordinated Regulation of Stomatal and Root Development

**DOI:** 10.1371/journal.pone.0156930

**Published:** 2016-06-08

**Authors:** Christopher Hepworth, Carla Turner, Marcela Guimaraes Landim, Duncan Cameron, Julie E. Gray

**Affiliations:** 1 Department of Animal and Plant Sciences, University of Sheffield, Sheffield, United Kingdom; 2 Department of Molecular Biology and Biotechnology, University of Sheffield, Sheffield, United Kingdom; National Taiwan University, TAIWAN

## Abstract

Root development is influenced by nutrient and water availabilities. Plants are able to adjust many attributes of their root in response to environmental signals including the size and shape of the primary root, lateral roots and root hairs. Here we investigated the response of roots to changes in the levels of leaf transpiration associated with altered stomatal frequency. We found that plants with high stomatal density and conductance produce a larger rooting area and as a result have enhanced phosphate uptake capacity whereas plants with low stomatal conductance produce a smaller root. Manipulating the growth environment of plants indicated that enhanced root growth is most likely a result of an increased demand for water rather than phosphate. Plants manipulated to have an increase or reduction in root hair growth show a reduction or increase respectively, in stomatal conductance and density. Our results demonstrate that plants can balance their water uptake and loss through coordinated regulation of both stomatal and root development.

## Introduction

Recent advances in molecular genetics provide the resources to dissect the effect of water loss on plant growth and performance. Work in the genetic model species *Arabidopsis thaliana*, to unravel the molecular basis of stomatal development, has resulted in a series of mutants which range in stomatal density (*D*), stomatal conductance, transpiration (*E*), water use efficiency and carbon assimilation in the same genetic background [1–4]. Arabidopsis plant lines with either low or high *D* have been produced by manipulating the level of expression of EPIDERMAL PATTERNING FACTORS (EPF). In the current study we focused on genotypes with manipulated levels of EPF1 and EPF2; peptides which normally act to suppress inappropriate stomatal development. Plants manipulated to ectopically overexpress *EPF2* have substantially reduced *D* on leaves, and in our previous study, had approximately 50% of the *E* of wild-type mature leaves [5]. In contrast, *epf1epf2* double mutant plants, lacking both *EPF1* and *EPF2* expression, have a high *D* and approximately 170% of the *E* of wild-type leaves.

Here we investigated direct uptake of phosphate (*P*) by the roots of plants with manipulated stomatal densities. *P* is an essential plant nutrient which is actively taken up by transporters predominately located on the surface of root hairs. A relationship between *D* and nutrient accumulation via mass flow has been previously reported [5]. We sought to address whether this relationship extended to direct nutrient uptake via the roots by examining the accumulation of a radioactive phosphate isotope, supplied to the roots as ^33^P-orthophosphate. Furthermore, It is generally accepted that the presence of root hairs is important for both water and nutrient uptake by increasing the extent of the rhizosphere and maximising the root to soil interface. Root hairs may also act as environmental sensors in particular of water stress [6]. The molecular genetic basis of root development has been well studied [7] and in particular a family of transcription factors including ROOT HAIR DEFECTIVE 6-LIKE 4 (RSL4), ROOT HAIR DEFECTIVE 6 (RHD6) and RHD SIX LIKE1 (RSL1) have been shown to regulate root hair patterning/development [8, 9]. Plants lacking the expression of these genes display shorter root hairs whilst those overexpressing *RSL4* develop longer root hairs.

To our knowledge no study has previously compared the root structure or phosphate uptake between plants of the same species with significantly differing levels of *D* or *E g*rown under the same conditions. Likewise it is currently unknown what effect altering root hair development may have on *D* or *E*. Via manipulation of both the stomatal and root hair development pathways, we sought to address these questions.

## Results and Discussion

**Fig 1:**
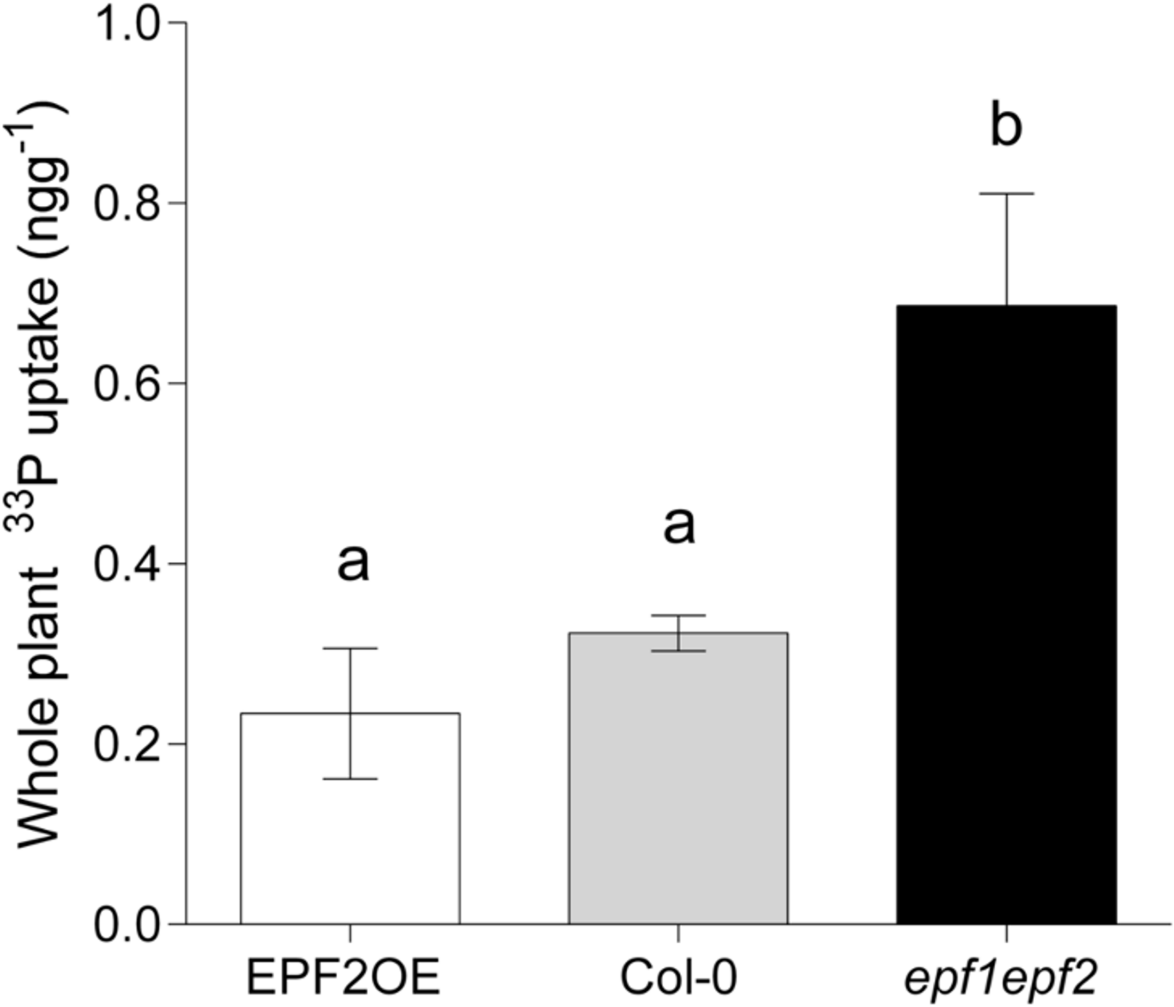
Increasing stomatal density enhances *P* accumulation. Mean total [^33^P] concentrations (ng g^−1^) taken up directly by whole plants over 5 hours (n = 5). Bars with no letters in common are significantly different, P<0.05, (Tukeys test after one-way ANOVA). Error bars represent SE.

Five hours after transfer of *Arabidopsis thaliana* seedling roots to an aqueous nutrient solution, plants with high *D* (*epf1epf2*) had taken up over twice as much radioactive phosphate as wild-type. Low *D* plants, (the *EPF2*OE genotype) showed only a slight reduction in phosphate (*P*) accumulation, and this was not significantly different to the level of *P* taken up by wild-type plants under the same conditions (Fig 1). This suggested that, whilst an increase in *D* and *E* may promote *P* accumulation the opposite did not hold true; a large decrease in *E* had no significant effect on *P* uptake.

**Fig 2:**
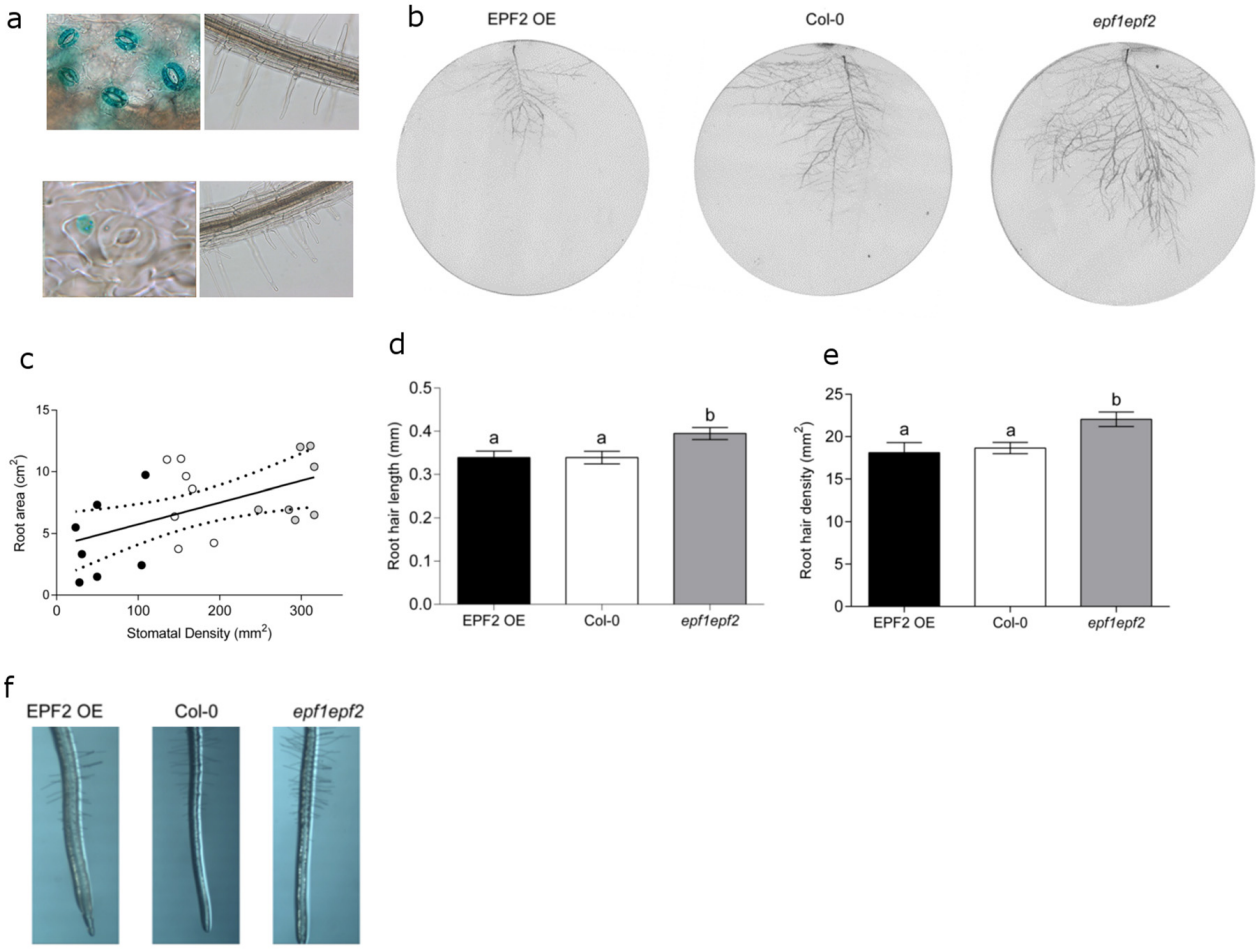
Stomatal density correlates with root area. (*A*) Histochemical staining of seedlings expressing *pEPF1*::*GUS* (top) indicates *EPF1* expression in guards cells but not in roots, and *pEPF2*::*GUS* (bottom) indicates *EPF2* expression in early stomatal lineage cells and not in roots. (*B*) Representative scans to illustrate root size of plants grown in rhizotrons. (*C*) Linear regression showing a significant relationship between stomatal density and the rooting area of plants grown in rhizotrons (P = 0.0083). (*D*) Increased root hair length in plants with increased stomatal density. (*E*) Increased root hair density in plants with increased stomatal density. (*F*) Representative micrographs of plants grown in agar to assess root hair length and density. Bars with no letters in common are significantly different, P<0.05, (Tukeys test after one-way ANOVA). Error bars represent SE.

We then investigated whether the increase in *P* accumulation seen in plants with increased *D* could be a result of an increased root surface area for nutrient uptake. First we sought to confirm that *EPF1* and *EPF2*, which have previously been reported to be expressed in immature stomata and stomatal precursor cells [10–12] do not have a direct effect on root anatomy and physiology. Histological staining of plants expressing β-glucuronidase (GUS) under the control of either the *EPF1* or *EPF2* gene promoter regions (Hunt & Gray, 2009) indicated that neither of these genes are expressed in seedling roots (Fig 2a) which is in line with transcriptomic studies [13]. Thus, it appears unlikely that the absence of *EPF1* and *EPF2* expression in the *epf1epf2* mutant could have a direct effect on the roots. It is of course possible that, these secreted peptides could travel to the roots, or that the constitutive over-expression of *EPF2* in the *EPF2*OE mutant could have a direct effect on root nutrient uptake. Indeed EPFL9, a positive regulator of stomatal development, has been previously shown to have a role in root cortex development [14]. However, no significant differences in *P* accumulation were observed in the EPF2 over-expressing genotype (Fig 1).

We examined the roots of our plants in several different ways. Each of our measurements suggested that plants with increased *D* had an increased root area. First we grew plants in rhizotrons with their roots supported on glass fibre disks. After 7 weeks when the leaf rosette was fully expanded we scanned the glass fibre disks to assess root area, and took leaf impressions to record *D*. A linear relationship was found suggesting a positive correlation between root area and *D* (Fig 2b and 2c). Next, we examined the root hairs of young plants grown on nutrient agar plates. We found that both the length and density of root hairs were significantly enhanced in plants with increased *D* (*epf1epf2*) in comparison to wild-type whilst low *D* plants (EPF2OE) shown no root hair phenotype (Fig 2d, 2e and 2f). Together these results indicated that plants with high *D* and thus an increased capacity for water-loss also displayed increased root area and root hair growth. Since the active transport of *P* occurs primarily at the root hairs this result, could at least in part, explain the enhanced *P* accumulation that we observed in the *epf1epf2* mutant (Fig 1).

**Fig 3:**
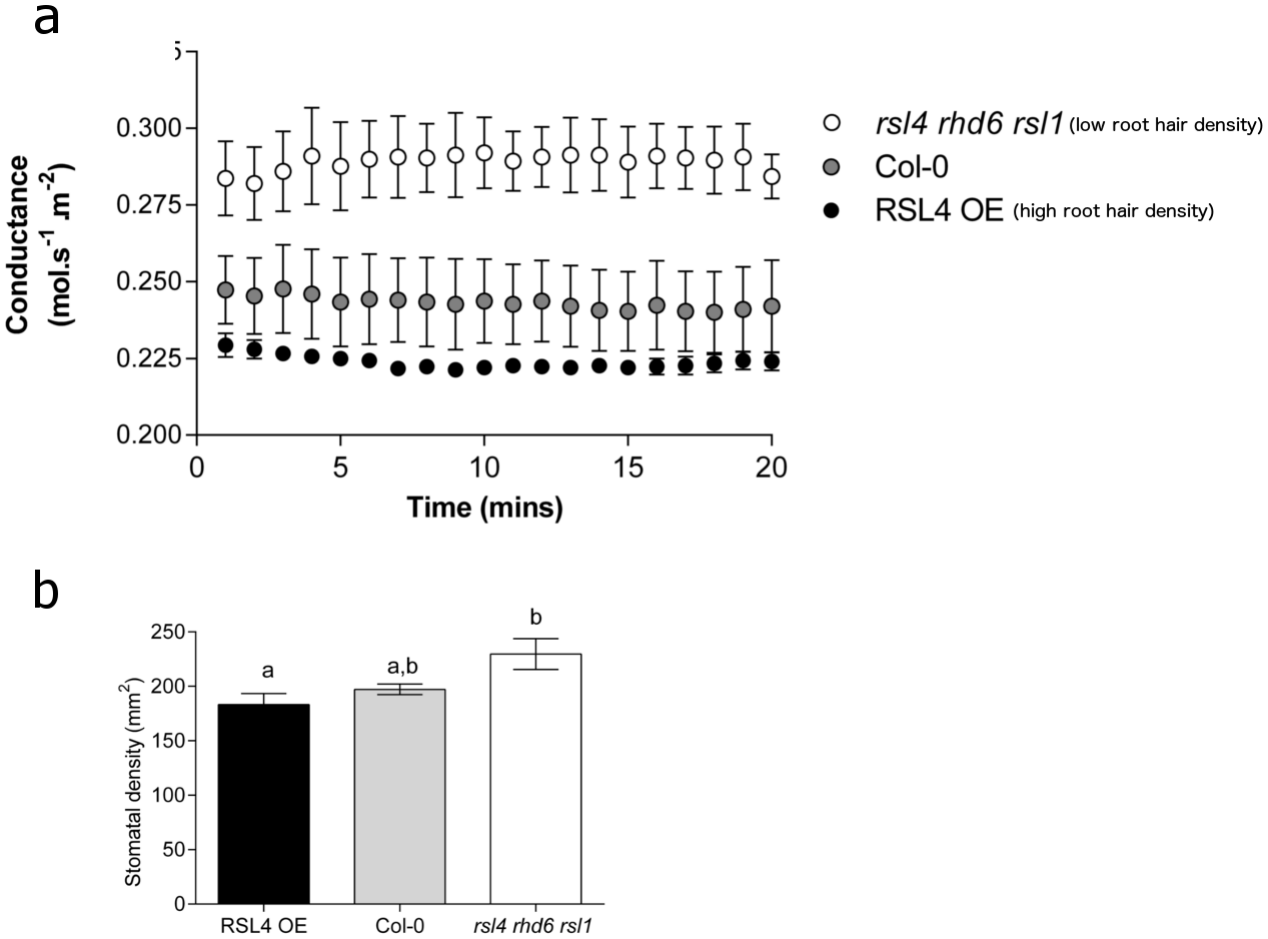
Plants with altered root hair density and length display correspondingly opposite alterations in stomatal conductance and stomatal density. (*A*) Mean steady state stomatal conductance rates for genotypes with decreased (*rsl4rhd6rsl1*; white symbols), or increased root hair length and density (*RSL4*OE; black symbols) in comparison to controls (Col-0; grey symbols) at 1000 μmol.s^-1^m^-2^ light, 400 ppm CO_2_ over 20 minutes. (*B*) Mature leaf abaxial stomatal densities of RSL4 overexpression or *rsl4rhd6rs1* genotypes. Bars with no letters in common are significantly different, P<0.05, (Tukeys test after one-way ANOVA). Error bars represent SE.

To further investigate the links between *D*, *E*, root architecture, and *P* accumulation, we carried out the converse experiment to examine whether plants with altered root hair growth exhibited any alteration in their stomatal characteristics. Remarkably, we found that plants specifically manipulated to have an increased density of root hairs (*RSL4*OE) [8] had significantly lower stomatal conductance values than a mutant manipulated to have low root hair density (*rsl4rhd6rsl1*) (Fig 3a). These plants were then assayed for difference in *D* and, as with stomatal conductance, *D* was inversely related to root hair growth (Fig 3b). It is unlikely that the alteration in stomatal development observed in the root hair mutants occurs as a direct result of gene mis-expression, as the *RSL1*/*RSL4/RHD6* genes do not appear to be expressed in the shoots of *Arabidopsis thaliana* and the overexpression of the *RSL4* gene does not alter shoot morphology [8, 15].

Our results reveal a previously undescribed interplay between *D* and rooting area that appears to have a functional effect on *P* accumulation. The exact mechanism and stimuli for these observations is currently unclear. It appears that the high rate of water loss found in *epf1epf2* plants may stimulate root growth in an attempt to maximise water uptake and as a result enhances *P* uptake capability. This may allow *epf1epf2* plants to be intrinsically more capable of accumulating nutrients both through increased rates of mass flow as in Hepworth et al.,2015 and through direct root interception.

The enhanced root growth phenotype we observe in *epf1epf2* plants is reminiscent of roots grown under *P* starvation [16]. Thus an alternative explanation might be that the *epf1epf2* plant has an enhanced requirement for *P* and therefore produces more root hairs. The additional guard cells produced in *epf1epf2* may require additional energy in the form of ATP to power their turgor-driven aperture changes [17]. It is therefore not clear whether the enhanced *P* uptake capability that we observed in *epf1epf2* plants is due to their enhanced *E* levels necessitating a higher water uptake capacity and/or a higher requirement for *P* in these plants.

**Fig 4:**
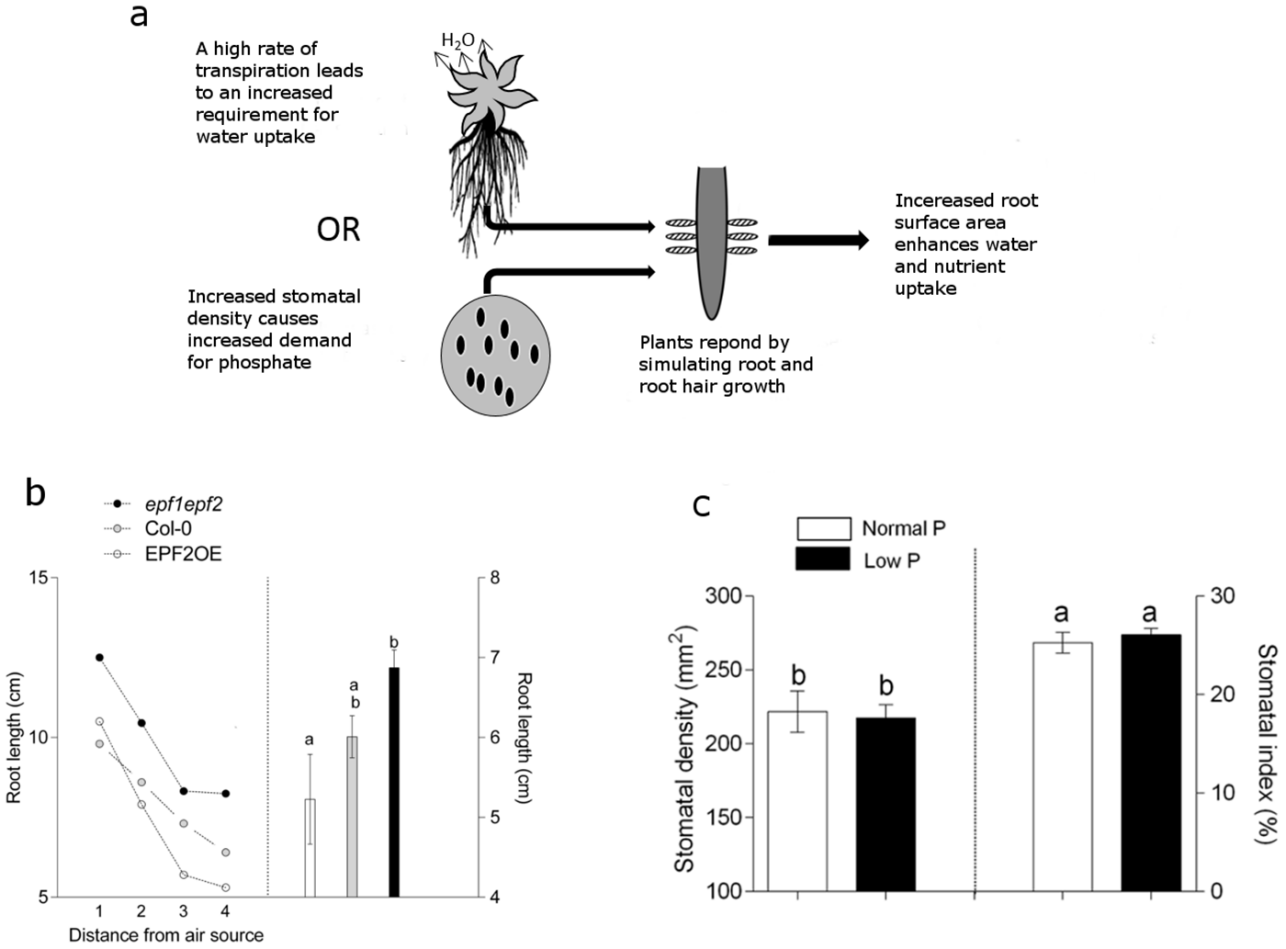
The promotion of root hair development is more likely to be due to an increased capacity for water loss rather than an enhanced demand for phosphate. (*A*) Two proposed models to explain the enhanced root development and phosphate accumulation observed in the *epf1epf2* mutant. (*Top*) Increased water loss promotes root hair development. (*Bottom*) increased frequency of stomata incurs a phosphate cost which leads to phosphate stress and the subsequent promotion of root hairs. (*B*, *Left)* A relationship exists between conditions which reduce leaf boundary layer resistance and rooting length. The X axis denotes distance from a moving air source with 1 being the closest. (*B*, *Right*) Plants grown under identical conditions without the presence of a moving air source. A relationship between transpiration/stomatal density and rooting length exists. (*C*) Plants grown under phosphate limiting conditions display normal levels of stomatal density. Bars with no letters in common are significantly different, P<0.05, (Tukeys test after one-way ANOVA). Error bars represent SE.

To investigate these two possibilities (Fig 4a), we first grew plants under conditions that promoted different levels of *E* by altering the distance of plants from a moving air source (a rotating fan) thus manipulating airflow at the height of the leaf boundary layer. We found that plants grown under growth conditions which promoted *E* (those closest to the fan) produced the longest roots and those sheltered from the moving air produced the shortest roots (Fig 4b). The trend towards larger roots under fast moving air was evident across all three genotypes; plants with low, normal or high *D* increased their root length by 40%, 33%, and 30% under fast moving air in comparison to the same genotypes grown normally (Fig 4b *left*).

In a separate experiment, plants were grown under two different *P* availabilities, the lower of which significantly limited growth (S1 and S2 Figs). The *D* of plants at growth limiting levels of *P* availability was unaffected indicating that limiting *P* availability does not impair stomatal development in *Arabidopsis thaliana* (Fig 4c). These results suggest that the driving force for increased root growth in high *D* plants is more likely to be to accommodate for high levels of water loss, than to satisfy a demand for high levels of *P* needed for stomatal development and function as previously suggested [2, 18]. The nature of the shoot-to-root, and root-to-shoot, signals which we propose act to balance *E* and water uptake capacities are currently unknown. However plant hormones, including abscisic acid which is well known to modulate root architecture [19–21] stomatal conductance [22] and more recently stomatal development [23, 24] are obvious candidates.

This study has demonstrated that plants manipulated to have high maximal *E* respond by promoting root and root hair growth which enhances *P* accumulation, whereas those manipulated to have a larger rooting area, (and potentially increased nutrient and water uptake capacity), adapt their development to reduce stomatal conductance and *D*. We also show that in the case of *EPF2*OE, it is possible to reduce stomatal development to an extent that water use efficiency is substantially increased [1], nutrient accumulation is not impaired [5] and root hair development is not significantly altered (Fig 2). Moreover, our results indicate that attempts to enhance yield, carbon assimilation or plant cooling through increasing water loss, may indirectly promote root growth, and therefore increase water and nutrient uptake capacities.

## Methods

### Plant Growth

All plant genotypes were in Col-0 background and have been previously described; stomatal development mutants by Hunt & Gray 2009 and root hair density mutants by Yi et al 2010. Seeds were stratified in the dark for 72 hours at 4°. Plants were grown in environmentally controlled growth chambers (Conviron BDR16) at 22°C/16°C, 9 hours light, 15 hours dark. To examine root hairs, plants were grown on 0.5x MS nutrient agar under sterile conditions. Plants used in Fig 1 were germinated on 0.5x MS agar before being transplanted into autoclaved sand and watered every two days with 20% Long Aston’s solution [25] until 21 days old. For growth under low or normal phosphate availabilities (Fig 4c); 20% Long Aston’s solution was prepared containing phosphate concentrations of 0.34mM or 0.034mM for normal and low phosphate conditions respectively. Plants were subjected to differing levels of area flow (Fig 4b) by positioning a fan (LLOYTRON F 039 12”) 15cm from the first row of plants. Each row contained 1 plant of each genotype at an exact distance of 7cm between each of 4 plant rows. An identical collection of plants were grown in an adjacent position but were shielded from the movement of air. All plants were rotated every day within their rows (but not between rows) and kept at a fixed distance away from the moving air source.

### Stomatal Density

Dental resin (Coltene Whaledent, Switzerland) was applied to the abaxial surface of a fully expanded leaf and left to dry for a minimum of 5mins. Dry dental resin prints were removed from the leaf and nail vanish applied to the resin where the leaf is at its maximum width. Stomatal counts were determined by light microscopy (Olympus BX51) on the nail vanish peels and images recorded with a digital microscope eyepiece (HiROCAM MA88-300A 3.0 Mega Pixels). A minimum of 3 areas per leaf, 3 leaves per plant and 3 plants per genotype were used for analysis.

### Phosphate accumulation

Holes were made in the lids of 55mm diameter petri dishes; one larger hole so that the roots of the plants could be placed inside the dish with the rosette remaining outside, and a second smaller hole for introducing radioactive orthophosphate into the solution in the base of the dish surrounding the roots. A total of 0.5MBq H_3_PO_4_ (specific activity 148 TBq mmol^-1^) in 1ml dH_2_0 was injected into the smaller hole along with 3ml of 20% long Ashton’s solution and both holes were then sealed. At this point the rosette was outside of the petri dish and not in contact with the orthophosphate solution. 5 plants of each genotype and 3 blank controls (dishes containing radioactivity but no plant) were assayed using identical Petri dishes, radioactive solution and sealed in the same manner. After 5 hours, plants were carefully removed from dishes, excess solution was fully blotted onto absorbent paper before material was dried, harvested and digested. Radioactivity and *P* content was quantified by liquid scintillation counting as in Field et al 2012. Readings were corrected for decay and subtracted from blanks before absolute phosphorus was calculated.

### Histochemical Staining

*EPF1*::*GUS* and *EPF2*::*GUS* promoter reporter expressing plants (Hunt and Gray 2009) were grown vertically on agar for ten days, removed, placed in X-Gluc reagent (50mM potassium phosphate, 1mM potassium ferrocyanide, 1mM potassium ferricyanide, 0.2% triton x-100, 2mM 5-bromo-4-chloro-3-indolyl-b-d-glucuronic acid and 10mM EDTA) and vacuum infiltrated for 15 minutes. Samples were incubated 37°C in the dark for 3 hours, cleared with 70% ethanol and images captured with an Olympus BX51 microscope connected to a DP51 digital camera.

### Rhizotron analysis

10 day old seedlings were transferred from 0.5x MS agar plates to rhizotrons consisting of a 150mm diameter petri dish filled with vermiculite, and a rock wool plug placed over the 1cm^2^ holes at the top and bottom of the dish. Glass microfibre paper (Whatman) was placed between the vermiculite and the plastic of the petri dish. Seedlings roots were placed between glass fibre paper and the plastic base of the petri dish with the shoot placed through the hole at the top of the petri dish. The dish was wrapped in tin foil so that the roots were in darkness and positioned vertically. Seedlings were watered biweekly with 0.25x MS solution. Rhizotrons were opened after 5 weeks and the glass fibre disk with roots attached scanned and analysed using GIMP and ImageJ software.

### Root Hair Measurements

Four Arabidopsis Col-0 plants were sown alongside 4 mutant plants, stratified, and grown vertically on 0.5x MS agar in 120mm x 120mm square Petri dishes. Root hair length and density were determined from a region 1cm above the first root hair from the root tip by light microscopy (Olympus BX51) and images taken with digital microscope eyepiece (HiROCAM MA88-300A 3.0 Mega Pixels) while still attached to the agar plates. At least 3 plates per genotype were measured.

### Infra-red Gas analysis

Measurements were taken with a LI-6400 portable photosynthesis system (Lincoln, NE) on mature, fully expanded leaves of 4 plants per genotype. Relative humidity was kept at 65%-75% using self-indicating desiccant, flow rate was 500μmol s^-1^ and block temperature 20°C. Reference CO_2_ was set to 450ppm, light intensity to 1000 μmol.m^-2^.s^-1^ and the plant left to equilibrate within the cuvette for at least 45 minutes. Measurements were taken every 30 seconds and the IRGA matched every ten minutes.

## Supporting Information

S1 Fig The leaf area of plants grown under non-limiting or limiting concentrations of phosphate. (TIF)

S2 Fig Digital pictures of plants grown under non-limiting (a) or limiting (b) concentrations of phosphate. (TIF)

S3 Fig Stylised diagram of the experiment setup reported in Fig 4b. (TIF)

S1 Table Raw data used to generate the means and errors for the graphs presented in this study. (XLSX)
