# Supplementary figures and images for: Balancing Water Uptake and Loss through the Coordinated Regulation of Stomatal and Root Development
Source: PLoS One. 2016 Jun 8;11(6):e0156930. doi: 10.1371/journal.pone.0156930 (PMC4898744; doi:10.1371/journal.pone.0156930)

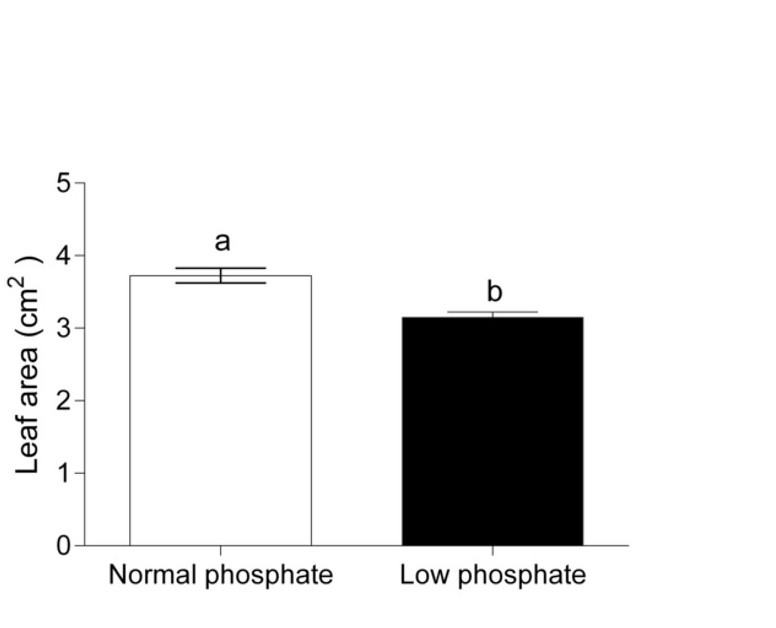

Supplement: S1 Fig — (TIF) [file pone.0156930.s001.tif]

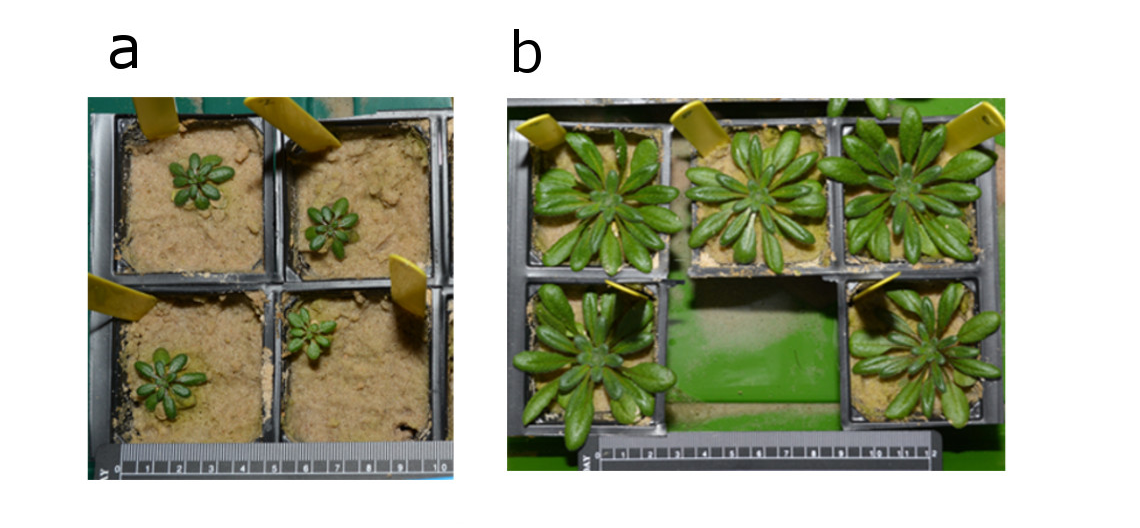

Supplement: S2 Fig — (TIF) [file pone.0156930.s002.tif]

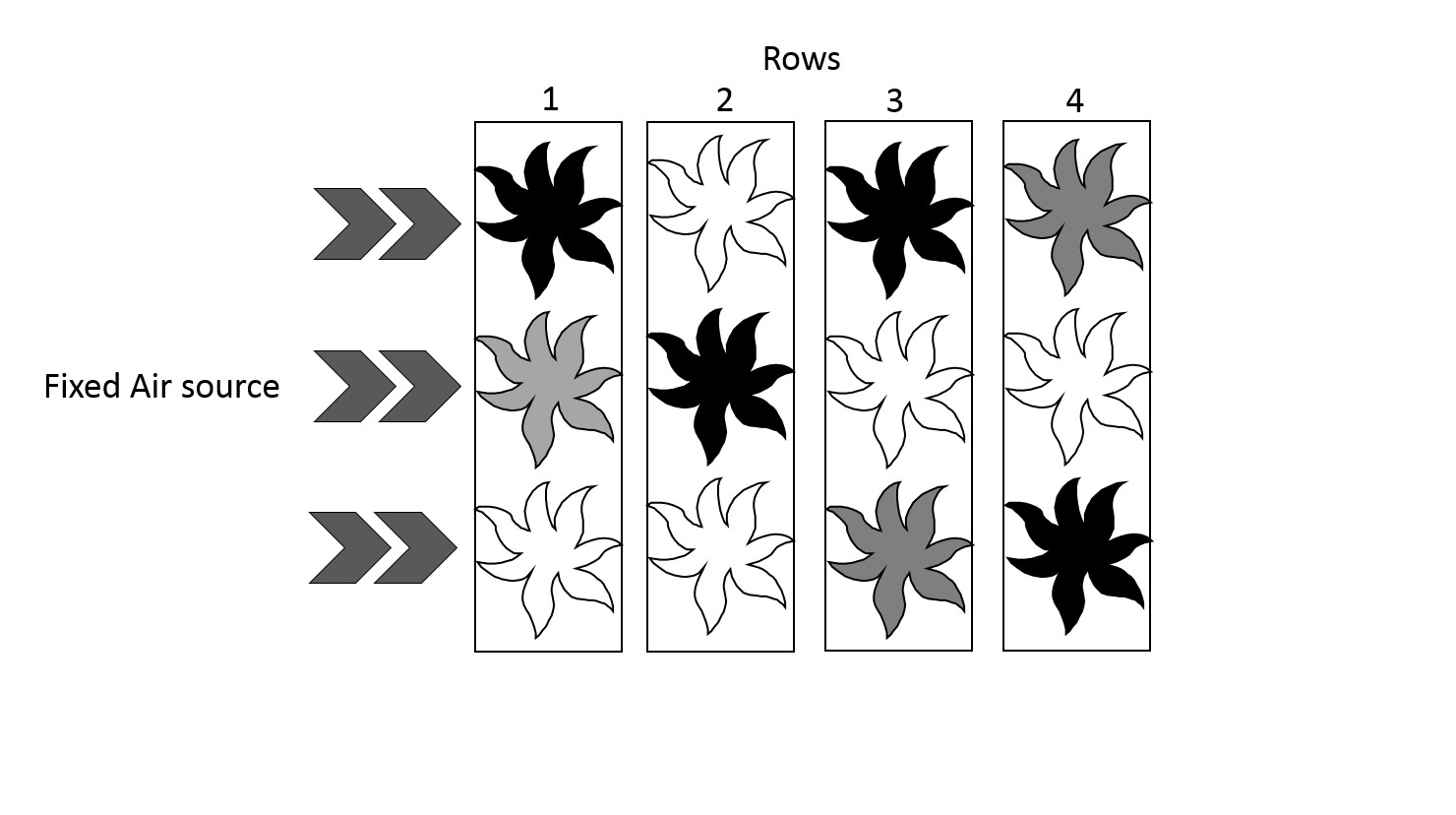

Supplement: S3 Fig — (TIF) [file pone.0156930.s003.tif]
